# Gut Microbiome Interactions with Oxidative Stress: Mechanisms and Consequences for Health

**DOI:** 10.3390/pathophysiology31030023

**Published:** 2024-06-21

**Authors:** Natalya Semenova, Nadezhda Garashchenko, Sergey Kolesnikov, Marina Darenskaya, Liubov Kolesnikova

**Affiliations:** Scientific Centre for Family Health and Human Reproduction Problems, 664003 Irkutsk, Russia; nadzelin@mail.ru (N.G.); sikolesnikov2012@gmail.com (S.K.); marina_darenskaya@inbox.ru (M.D.); kolesnikova20121@mail.ru (L.K.)

**Keywords:** gut microbiome, oxidative stress, food supplements, TMAO, melatonin, mitochondria

## Abstract

Understanding how gut flora interacts with oxidative stress has been the subject of significant research in recent years. There is much evidence demonstrating the existence of the microbiome–oxidative stress interaction. However, the biochemical basis of this interaction is still unclear. In this narrative review, possible pathways of the gut microbiota and oxidative stress interaction are presented, among which genetic underpinnings play an important role. Trimethylamine-N-oxide, mitochondria, short-chain fatty acids, and melatonin also appear to play roles. Moreover, the relationship between oxidative stress and the gut microbiome in obesity, metabolic syndrome, chronic ethanol consumption, dietary supplements, and medications is considered. An investigation of the correlation between bacterial community features and OS parameter changes under normal and pathological conditions might provide information for the determination of new research methods. Furthermore, such research could contribute to establishing a foundation for determining the linkers in the microbiome–OS association.

## 1. Introduction

The human gut houses a diverse bacterial community referred to as the microbiome [1]. The complex communities demonstrate host specificity in their composition and function and have co-evolved with their hosts. The gut microbiome structure is affected by a variety of factors, including the environment, diet, age, and geographical location [2,3,4,5,6]. It has been suggested that the number of human cells in the human body may be fewer than its microbial cells and that the number of human genes may be 10–100 times less than what is found in the intestine of the host [1,7,8,9]. In the last few decades, the amount of research conducted on the influence of the gut microbiome on the host organism, in a field called microbiomics, has increased significantly [10,11,12]. A new theory about the microbiome being a human “organ” gave impetus to investigating mechanisms of interactions between bacterial communities and other organism systems. Oxidative stress (OS) components may be involved in the interregulation of the microbiome and macroorganism and its regulatory mechanisms [13]. OS is caused by an imbalance in the oxidant/antioxidant system in favor of the oxidants, leading to disorganization in redox signaling and control and/or molecular damage [14]. A major mechanism of OS tissue damage is oxidant-induced apoptosis and necrosis caused by an increase in mitochondrial membrane permeability and the release of cell-destructing factors [15]. The antioxidant mechanisms (specific enzymes or combined biochemical processes) prevent cell damage and restore oxidative homeostasis [16,17,18]. OS is associated with many different diseases [19,20,21,22,23,24]; however, there are many gaps in our understanding of the mechanisms under normal and pathological conditions since the casual association between OS and the microbiome has drawn attention only recently. Revealing the mechanisms underlying the gut microbiome and OS interactions may pave the way for developing novel therapeutic strategies for different disorders.

## 2. The Cooperation of the Microbiome and OS in Disease Development and Other Conditions

The description of the human gut microbiome and its changes with host aging is one of the main issues discussed in the field of microbiomics. The research on fecal microbiomes from patients of different continents demonstrates the possibility of the identification of health-aging changes in bacterial communities [3]. Moreover, the disease-associated taxa are identified quite often [25,26].

The abnormalities in gut microbiome and OS development are identified in a variety of disease studies [27,28,29]. These two system changes are being evaluated in inflammatory disorders such as inflammatory bowel disease (IBD), liver inflammation, acute pancreatitis, and osteoarthritis [13,30,31]. A large number of results have been obtained from intestine inflammation models [13,32,33,34,35,36]. IBD is a complicated and multifactorial group of disorders characterized by relapsing and remitting inflammation. Crohn’s disease (CD) and ulcerative colitis (UC) are the two types of IBD [37]. OS is considered a main factor in the pathogenesis of IBD and might be an important participant in cell damage and tissue injury processes [38]. This is supported by the results of a colitis modeling study using dextran sulfate sodium. In one study, glutathione peroxidase (GSH-Px) and superoxide dismutase (SOD) activities, as well as glutathione (GSH) content, decreased, whereas malondialdehyde (MDA) levels increased in the disease-modeled group compared with the control group [36]. In another study on experimental IBD in a mouse model, similar alterations were detected; in addition, total antioxidant capacity (AOC) and catalase (CAT) decreased significantly, and the level of reactive oxygen species (ROS) was significantly higher in the disease group compared with the control group [33]. Similar perturbations were reported in the disease groups of both studies. The relative abundances of Bacteroidetes and Cyanobacteria were decreased, and the relative abundance of Firmicutes was increased in the disease group compared with the control group. The relative abundances of Lachnospiraceae and Ruminococcaceae were decreased, and the relative abundances of Bacteroidaceae, Clostridiaceae, Lactobacillaceae, and Turicibacteraceae were increased in the disease group compared with the control group [36]. *Bacteroides*, *Turicibacter*, *Actinobacteria*, *Acholeplasmatales*, *Eubacterium*, *Staphylococcales*, *Enterobacterales*, and *Rhodespirillales* predominated in the disease group [33].

Interestingly, similar OS parameters results were observed in a Cd-induced liver inflammation model: GSH-Px and SOD activities decreased, whereas MDA and ROS production increased in the disease group compared with the control group in first-stage experiments and fecal transplantation tests. However, there were no significant changes in the antibiotic-treated groups [31]. This demonstrates a possible link between OS and alterations in the gut microbiome.

**Figure 1:**
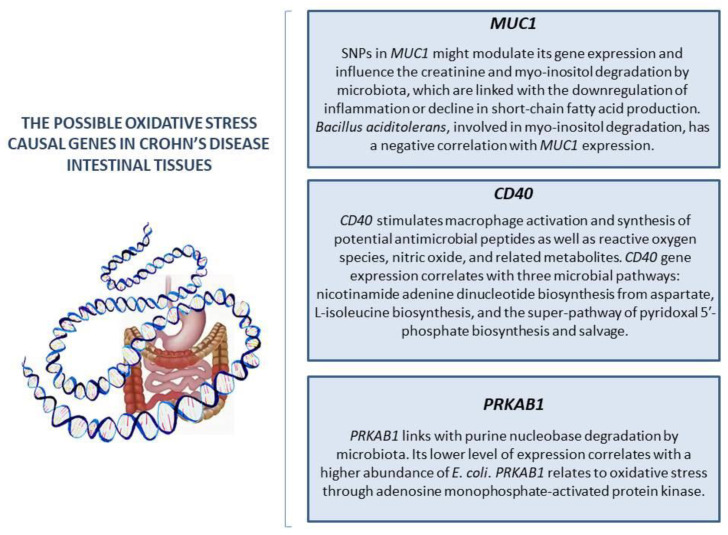
The possible oxidative stress causal genes in Crohn’s disease intestinal tissues.

IBD is often researched from a genetic point of view as well [39]. Many genes control OS development [40,41]. Unlike many other processes, the relationship between genes and OS is bilateral. In one respect, genes control the synthesis of antioxidants and any changes; therefore, mutations may lead to abnormalities in gene expression. Moreover, mitochondrial DNA could also be damaged, which might affect ROS production. On the other hand, this damage could be provoked by OS itself. Moreover, OS may negatively influence the proliferation of cells [16,18,40,41,42]. The genetic foundation of the interconnection between the microbiome and OS has also been investigated. Mucosal SOD isoform expression has been found to be differentially modified in IBD patients [43]. In that study, the MDA concentration was elevated in both CD and the ulcerative colitis mucosa. In the UC group, the amount of MDA was associated with epithelial CAT expression and neutrophilic myeloperoxidase activity. On the other hand, the CD group is associated with the concentration of Mn-SOD activity [44]. Furthermore, Mn-SOD protein levels increased in patients with IBD; however, Cu/Zn-SOD decreased according to inflammation levels [43]. The genetic study of the role of OS in CD presented new data on the interactions between oxidation processes in organisms and microbiota. Xu et al. (2023) [45] tried to identify the hypothetical causal effects and molecular mechanisms of OS genes in CD (Figure 1). They presented hypoglycosylated abnormal mucin 1 (*MUC1)* as a main possible OS causal gene in CD intestinal tissues associated with the gut bacterial community. The authors assumed that single-nucleotide polymorphisms (SNPs) in *MUC1* might modulate its gene expression and influence the creatinine degradation and myo-inositol degradation by microbiota, which are linked with the downregulation of inflammation or decline in short-chain fatty acid (SCFA) production. The elevated expression of this gene is presented as a cause of CD. Moreover, a negative correlation between *Bacillus aciditolerans*, involved in myo-inositol degradation, and *MUC1* expression was observed in the control. Therefore, *MUC1* expression relates to microbiome structure; moreover, *MUC1* expression and bacterial myo-inositol and creatinine degradation share genetic effects. The authors suggested this fact points out the potential interactions between the gene and microbiota. The second gene studied is *CD40*. This gene stimulates macrophage activation and synthesis of potential antimicrobial peptides as well as ROS, nitric oxide, and related metabolites [46]. *CD40* gene expression correlates with three microbial pathways: nicotinamide adenine dinucleotide biosynthesis from aspartate, L-isoleucine biosynthesis, and the superpathway of pyridoxal 5′-phosphate biosynthesis and salvage. It was that these microbiota-derived substances are associated with intestinal inflammation. The third gene studied is protein kinase amp-activated beta 1 (*PRKAB1)*, which links with microbial purine nucleobase degradation. Its lower level of expression correlated with a higher abundance of *E. coli* in the disease group [45]. *PRKAB1* relates to OS through adenosine monophosphate-activated protein kinase (AMPK) effects since it encodes one of the AMPK subunit isoforms [47]. The authors suggested that the common genetic regulations of intestinal gene expression and bacterial metabolic potentials were observed. Nonetheless, the intestinal gene expression was not significantly colocalized with any individual taxa in the study. In the end, the authors argued that functional research might provide more information about underlying biological mechanisms [45]. However, genetic studies in this direction are complicated; thus, the biochemical consequences of genetic alterations require more attention.

The new concept of the Microbiota–Gut–Brain Axis presents a modern research area to investigate the pathological foundation of neurological diseases such as Parkinson’s disease and Alzheimer’s disease [28,48,49,50,51]. The key concern of this concept is the bidirectional communication between the gastrointestinal tract and the central nervous system [52]. Considering OS as one of the main mechanisms of brain tissue damage [53], another point raised in the research is the role of OS in the Microbiota–Gut–Brain Axis in human neurological disorders [49,50]. Potential therapeutic approaches have been described based on these relationships. Thus, it was demonstrated that probiotics can increase antioxidant enzyme activity levels in a Parkinson’s disease model and improve oxidative balance [51,54]. Therefore, understanding the role of OS in the Microbiota–Gut–Brain Axis is important for clinical purposes.

In addition, the microbiome and OS interaction in metabolic disorders [55,56,57,58,59,60], alcohol consumption [61], aging [62], and intestinal injury [63] have drawn attention. Although the number of studies in this field is rising every year, there are still many points to be clarified as well as unidentified molecules that might be relevant to this interaction. The possibility of the microbiome and OS interconnection affecting other organ and signaling systems in an organism in normal and pathological conditions is another important research line.

Aging and aging-related metabolic perturbations are serious issues for public health. Age-related alterations include gut microbiome transformation and oxidative system changes [64]. OS is associated with many different diseases and is considered a threat to healthy aging [16]. The common protocol for modeling aging includes D-galactose administration, which negatively influences the oxidative balance: the MDA level increases and antioxidant enzyme activity decreases. Therefore, as a result, the OS model for aging was developed. In serine deficiency conditions, oxidative balance deviations were observed to increase significantly [62]. Gut microbiome fluctuations were also mentioned, especially the Firmicutes/Bacteroidetes ratio downturn, which escalated in the serine deficiency group. However, the correlation between these processes was not tested [62,64]. Investigation of this link might be important for geriatric medical care.

Obesity is considered a serious issue for medical care systems around the world; therefore, the investigation of the relationship between the bacterial community and OS under this condition might be relevant for clinical care [58]. In the research presented by Hu et al. (2021) [56], maternal obesity on the gilt model was investigated. The authors assumed that obesity may influence placentae OS and gut microbiota composition. They demonstrated a positive correlation between Proteobacteria and the concentrations of ROS and protein carboxyl and a negative correlation with the concentrations of propionate and butyrate. Furthermore, a specific genus of Christensenellaceae and Ruminococcacea was positively correlated with the concentration of protein carboxyl; on the other hand, Bacteroidetes negatively correlated with the concentrations of placental ROS and protein carboxyl. However, the mechanisms of the link were not discussed. The next research team carried out a pilot study in adolescent cohorts analyzing variations in the microbiome community of patients with obesity and normal weight. Teenagers with normal weight were classified into one group based on the phylogenetic diversity of the active microflora. The other participants with active microflora were divided into three different groups and partly in the first one. In contrast, the β-diversity of all patients showed no differences in the main and control cohorts [57]. The parameters of OS in adolescents with obesity were also estimated in another study in a separate group. In the study, the OS index was approximately seven times higher in the group with obesity than in the control group. Moreover, diene conjugates, α-tocopherol, and retinol concentration, as well as SOD activity, were lower in the main group compared with the control one, however the link between microbiome diversity and OS was not investigated [59].

Another research group studied OS parameters and intestinal microbiome in the metabolic syndrome cohort. According to their results, oxidation products such as MDA and 8-hydroxydeoxyguanosine (8-OHdG) were significantly higher in a group of patients with metabolic syndrome. The authors pointed out that vitamin E and zinc levels were also lower in the patients’ cohort than in the control. The number of opportunistic pathogenic microflora markers was higher, and the number of normal microflora markers was less in the metabolic syndrome group. Moreover, in this group, *Eubacterium lentum* markers were significantly elevated, and *Clostridium hystolyticum* and *Nocardia* markers were doubled. Moreover, *Clostridium propionicum*, *Bacillus cereus*, *Prevotella*, and Enterobacteriaceae, which were not identified in the control group, were detected. The authors added that there were no significant fluctuations in the number of *Lactobacillus* markers. At the end of the article, the authors reported the hypothesis that OS causes dysbiosis characterized by opportunistic pathogenic microbiota overgrowth [60].

Ohira et al. (2021) [61] have studied how alcohol consumption, gut health, oxidants, and the microbiome may be related. The authors stated that, in previous works, the rise in ROS production in gut cells caused by ethanol oxidation had been discussed. They studied the gut bacterial communities of alcoholic and non-alcoholic people to characterize the effect of alcoholism on the human gut microbiome. In the study, dysbiosis was determined to be a result of alcohol intake. In this group, obligate anaerobe (such as *Bacteroides* and *Ruminococci*) decline was observed, and conversely, facultative anaerobe (such as *streptococci* and bacterial species belonging to Enterobacteriaceae) growth was recorded. The authors argued that these results are in line with the proposed sustained formation of ROS and ROS-induced OS in the colonic environment during chronic ethanol consumption since obligate anaerobes are generally more susceptible to ROS than facultative anaerobes [65]. The authors then investigated the effects of chronic ethanol administration on the fecal microbiome and colonic OS in a mouse model. They demonstrated fluctuations in 8-OHdG level in the colon, 4-hydroxynonenal (4-HNE) level upturn after just 10 weeks of the highest dose administration, and stability in nitrotyrosine level. Interestingly, the 8-OHdG level picked up after two weeks of ethanol intake, but it decreased eight weeks later. Unfortunately, in gut microbiota structure tests, the last control point is not 10 weeks but 5. The α-diversity showed no difference between groups; however, the bacterial community structure began to change. The growth of Bacteroidetes and decline in Firmicutes and Deferribacters in the microbiome of the ethanol groups was demonstrated. In the highest dosage ethanol group, Proteobacteria demonstrated an upturn, and in the lowest dosage group, there were fewer Deferribacters. Disappointingly, the possible correlations between OS parameters and microbiome alterations were not tested. The underlying mechanisms were not discussed as well [61].

**Figure 2:**
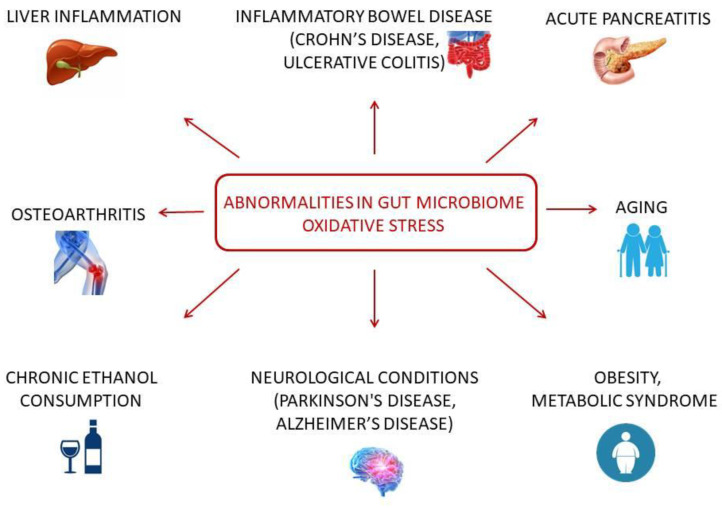
Diseases and conditions in microbiome-OS cooperation.

As can be noted from all these examples, common patterns in both changes are registered; however, a correlation between these changes is quite rare. Many different diseases, conditions, and factors can influence all these processes, so it is important to study them all (Figure 2); however, the more important research direction is uncovering the mechanism of such processes and transformations. The investigation of molecules that could participate in the interconnection between OS and microbiome under normal and pathological conditions might provide useful information to improve quality of life.

## 3. Possible Interconnections between OS and the Microbiome

### 3.1. Trimethylamine-N-Oxide (TMAO)

**Figure 3:**
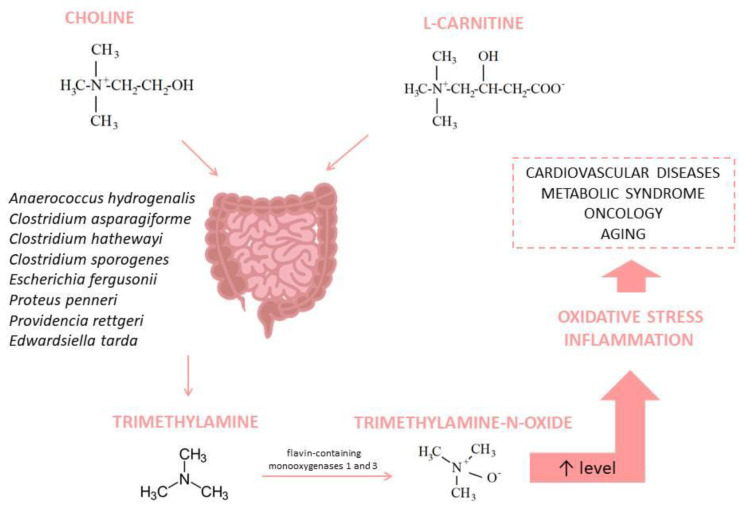
Trimethylamine-N-Oxide synthesis and its association with diseases.

Several hypotheses could explain the existence of a correlation between the gut microbiome and OS, wherein the TMAO mediation is one of them. Choline is an important compound for cell membrane structure; it plays a role in cholinergic neurotransmission and methyl group donation [66]. Choline is metabolized by gut microbiota into trimethylamine (TMA) [67]. L-carnitine is also a source of TMA in the gut [68]. Many bacterial species produce TMA; for example, according to the results of a study, two phyla (*Firmicutes* and *Proteobacteria*) and several genera showed significant choline consumption and TMA production: *Anaerococcus hydrogenalis*, *Clostridium asparagiforme*, *Clostridium hathewayi*, *Clostridium sporogenes*, *Escherichia fergusonii*, *Proteus penneri*, *Providencia rettgeri*, and *Edwardsiella tarda* [69]. After absorption, flavin-containing monooxygenases 1 and 3 (FMO1 and FMO3) transform TMA into TMAO in the host liver [70]. The elevated level of TMAO is associated with several disorders, including cardiovascular diseases, oncological problems, and metabolic syndrome [67]; it also promotes inflammation and OS [71] (Figure 3).

In a mouse model, the TMAO level was significantly higher in the old group, and the authors suggest it might be a consequence of aging-related gut dysbiosis. Moreover, they demonstrated the role of TMAO in aging-related OS in vessels [72]. Similar results were obtained in a study by Brunt et al. (2019) [73]. That study investigated age-related arterial dysfunction. Young and old mice were treated with antibiotics, and the TMAO level in the old control group was higher than that in the young control group. According to the results, antibiotics decreased TMAO in both groups. The changes in the gut microbiome were as follows: the phylum Proteobacteria predominated in treated groups mostly because of a significant increase in unclassified species within the family Enterobacteriaceae; the relative abundances of Firmicutes, Bacteroidetes, Deferribacteres, and Tenericutes were lower in treated groups compared with the controls; OS markers were higher in the old control group than in the young control group but fell after antibiotic therapy; and isoforms of superoxide dismutase were age-independent and antibiotics-affected or age-related but not affected by antibiotics. Unfortunately, the list of OS parameters in this research was quite short. Despite this, the authors pointed out that high TMAO levels associated with the changing microbiome composition might be a mechanism behind the microbiome–OS relationship.

Different studies have tested traditional Chinese medicines [74,75,76]. *Fructus Ligustri Lucidi* (FLL), which is used as an anti-aging medicinal plant to treat osteoporosis, was tested on mouse models. Also, OS parameter control and microbiome composition were observed. According to the results, FLL could prevent negative changes in microbiome structure in aging mice. Moreover, it caused circulating TMAO levels and OS to decrease. Based on the previous studies, the authors suggested an imbalance in microbiome composition; to be more precise, an increase in *Clostridium*, *Oscillospira*, *Sutterella*, *Desulfovibrio*, and *Coprococcus*, as well as a decline in *Bifidobacterium* and *Lactobacillus*, causes the rise in TMAO level, which, in turn, leads to OS. Therefore, in this article, TMAO is also considered as a possible link between the gut microbiome and OS [74]. However, there is no strong evidence that TMAO is a linker between the two processes. Possibly, it is an additional component of the OS development process.

### 3.2. Mitochondria and Short-Chain Fatty Acids

Another possible mechanism of interaction is mitophagy regulation. Omar et al. (2022) [77] stated that the gut microbiome may influence autophagy processes by producing SCFAs. SCFAs are the final products of microbial enzymatic transformation of dietary fiber. The bacterial species most involved in SCFA production are *Butyricicoccus* spp., *Faecalibacterium prausnitzii*, *Roseburia* spp., *Bacterioides* spp., and *Bifidobacterium*. SCFAs are also important for organs outside the digestive tract since many transmembrane proteins, receptors, and transporters that specifically bind SCFAs are expressed in a wide variety of cells [78,79,80,81]. It was stated that SCFAs may induce autophagy by inhibiting the mammalian target of rapamycin (mTOR) activity and downregulating AMPK activity. The elevated AMPKα phosphorylation was associated with cellular adenosine triphosphate (ATP) decline and ROS increase due to mitochondria [82]. The authors claimed that because the damaged mitochondria generate excess ROS, their autophagy leads to decreased OS in cells. The decline in SCFA-producing *Lactobacillus* spp., *Bacteroides* spp., *Prevotella* spp., *Streptococcus* spp., and *Phascolarctobacterium succinatutens* appeared to cause increases in Enterobacteriaceae and Clostridia. In addition, SCFA production was reduced. Therefore, the authors suggested that SCFAs might be a factor in how the gut microbiome is involved in oxidative processes in organisms [77].

In a study conducted in an acute pancreatitis model, the authors investigated the consequences of chitosan oligosaccharide administration. The disease was modulated by using injections of caerulein. According to the results, SCFA levels were noticeably higher in the group without acute pancreatitis that received treatment compared with the control group; moreover, the levels were higher in the control group that received treatment compared with the acute pancreatitis group that did not receive chitosan oligosaccharide treatment. As an antioxidant, chitosan oligosaccharide decreased OS (MDA in the treated group was lower than in the disease group, and SOD activity in the treated group was higher than in the disease group); however, it also influenced microbiome composition. First of all, it reversed the Firmicutes/Bacteroidetes ratio, which changed because of acute pancreatitis development. Furthermore, the abundance of the probiotic genera *Muribaculaceae* and *Akkermansia* increased. On the contrary, increases in proinflammatory *Desulfovibrio* and *Dubosiella* in the acute pancreatitis group were significantly lower in the cohort with disease and chitosan oligosaccharide administration. *Escherichia–Shigella* and *Enterococcus* showed the same tendency. Unfortunately, the association between OS parameters and microbiome features was not investigated. Interestingly, the results of the group that was administered with chitosan oligosaccharides without disease exhibited a significant increase in SCFA levels compared with the other groups. However, it barely correlated with the OS parameters and microbiome changes between the groups [32]. These results might point to the existence of complicated mechanisms including SCFAs as a linker between OS and the microbiome.

In a trial of other traditional Chinese medicines, *Lonicera hypoglauca* and *Scutellaria baicalensis* (plants rich in polyphenols), almost all OS parameters improved after the drug intake in a colitis mouse model. Extract administration also led to gut microbiota structural changes. The Firmicutes/Bacteroidetes ratio was higher in the treated group compared with the untreated group and the control, and other parameters presented several alterations in the untreated and control groups. Therefore, the relative abundance of *Alistipes* in the control group was considerably higher than that in the disease group, but in the treated group, *Alistipes* were even fewer. On the other hand, there were observable differences in the group treated with two other medicines in terms of *Dubosiella* and *Ruminococcus torques*. The relative level of *Lactobacillus* did not show a statistically significant difference. The SCFA levels in the colonic content were higher in the medicine-administered group in contrast with both groups. Unfortunately, the correlation between the OS parameters and microbiome compositions was not tested in this work [33].

Butyrate quite often draws the attention of researchers out of all the SCFAs. Butyrate is principally derived from the enteric microbiome. This compound might positively modulate mitochondrial function, including enhancing oxidative phosphorylation and β-oxidation and might support energy metabolism in unfavorable conditions by modulating the expression of several genes [1]. Important information was provided by a previously mentioned study in which the authors investigated OS and the microbiome in a Cd-induced liver inflammation model. They demonstrated the absence of changes in OS parameters in the Cd-induced liver inflammation and antibiotic-treated groups compared with the control group. The SCFA contents were estimated as follows: acetate, isobutyrate, and isovalerate showed no difference between groups, whereas propionate, butyrate, and valerate decreased in the disease group compared with the control group. However, there were no significant changes in the antibiotic-treated group. The correlation between the OS parameters and microbiome structure was not tested [31].

An additional statistical analysis of the results is summarized below to provide some data for future studies; however, disappointingly, the possibility of the existence of the OS–microbiome link is not well addressed in the literature, so various information could be lacking (Table 1).

### 3.3. Melatonin

Melatonin is considered a powerful antioxidant, and its deficiency can lead to the development of OS [83]. It is the main circadian rhythm regulator [84]. Melatonin is conventionally synthesized in the pineal gland through an enzymatic pathway from L-tryptophan. The gastrointestinal tract is considered a major source of extrapineal melatonin. However, its functions in the gut are not completely elucidated. It is suggested that melatonin regulates the motility of the lower gut and is involved in the regulation of gastrointestinal functions, including intestinal inflammatory processes [85]. It was demonstrated that melatonin and the microbiome appear to have a functional interconnection. The synthesis of melatonin in the gastrointestinal tract could be modulated by the bacterial community; moreover, melatonin is associated with the specific structure and changes in the gut microbiota [86,87,88,89]. It is suggested that melatonin might influence the gut microbiome, and an inverse relationship is also possible. According to the hypothesis presented by Zhang et al. (2021) [34], melatonin can regulate mitophagy in mouse liver partly because of its effects on the intestinal microbiota. The results showed that melatonin induced antioxidant enzyme activity and ameliorated mitophagy in the liver, reversing dysbiosis caused by mycotoxins. The authors drew attention to the fact that, after antibiotic treatment, melatonin administration did not influence mitophagy, which means that this is a microbiome-dependent mechanism. Tryptophan and its metabolites (melatonin is one of them) are also discussed as gut microbiome modifiers influenced by oxidative processes. Several bacteria transform tryptophan into its indolic derivatives, and some bacteria protect cells from oxidative damage [10]. Butyrate is one of the SCFAs produced by microflora, and in the OS condition, this compound counteracted the negative effect of ROS on tryptophan uptake [90].

In a study by Gao et al. (2021) [35], it has been shown that lack of sleep leads to corticosterone overproduction and dysbiosis, and excess corticosterone causes OS in organisms. Higher abundances of *Prevotella* and *Allobaculum*, as well as lower abundances of *Akkermansia*, *Bacteroides*, *Peptostreptococcus*, and *Lactobacillus*, were observed after corticosterone administration. The ratio of Firmicute/Bacteroidetes was also higher. Furthermore, the abundances of butyrate and tryptophan in this group were decreased. Melatonin administration reversed the changes provoked by sleep deprivation. The researchers suggested that mitochondrial function disturbances induce excess ROS and can affect the gut microbiota through normal intestinal environment disturbance, allowing bacterial antigens to penetrate the epithelium and provoke the immune response. Melatonin administration mitigated corticosterone levels in the mouse plasma. Along with the corticosterone decline, melatonin increased the colonic mitochondrial function, improved antioxidant defense, and normalized the intestinal bacterial community. The signaling pathways that undertake the discussed biochemical changes were also analyzed. In another study, it was proved that sleep restriction causes a rise in not only corticosterone but also norepinephrine and glucose. Furthermore, melatonin supplementation could reverse it. A similar situation was observed in the OS tests. The scientists reported the changes in the intestinal microflora in sleep-restricted mice. A higher abundance of harmful bacteria (*Helicobacter* and *Clostridium*) and a lower abundance of beneficial bacteria (Bacteroidetes and *Lactobacillus*) were observed in the jejunum. The mentioned dysbiosis was alleviated by melatonin supplementation. The authors assumed that OS improvement and inflammation inhibition caused by melatonin led to intestinal microbiota dysbiosis prevention in sleep-restricted mice [91]. These results might be used in support of the hypothesis of the existence of a complex system explaining the interconnection between OS and the microbiome including melatonin influence, SCFA effects, and mitochondrial processes. The investigation of the characteristics of this system may provide important information for theoretical science and practical application (Table 2).

## 4. Impact of Food Supplements on the Gut Microbiome and OS

Nutritional sports supplements can influence many different processes in organisms. Since they are now widely used, it is important to evaluate this influence. One of the most common ingredients is protein. Excess protein in food leads to several alterations in the organism [92]. According to a study carried out recently, there were no differences in MDA and SCFA levels between the group that received hydrolyzed beef and a whey protein supplement and the control group. However, after ten weeks of the experiment, the supplement intake group showed a higher abundance of Bacteroidetes and a lower abundance of Firmicutes; a lower presence of *Citrobacter* and *Klebsiella* was detected as well [93]. It is worth noting that only one OS parameter was tested, so the results of this study might not be sufficient to characterize the oxidative balance condition.

Methionine is an essential sulfur-containing amino acid for humans and animals. It demonstrates antioxidant properties and can be used as a protector against damage caused by several oxidants, including heavy metals. Thus, Wu et al. (2022) [94] reported that methionine can reverse intestinal OS and dysbiosis caused by nickel poisoning. It is noteworthy that even high levels of methionine groups quite often showed better oxidative conditions than the blank control. The slight difference in OS in segments of the intestine warrants attention. However, the authors did not mention this difference in their discussion and, instead, evaluated OS in the whole intestine. The microbiome changes were also tested in the whole intestine. As well as oxidative parameters, dysbiosis was reversed using methionine. The authors concluded that OS led to bacterial community changes. They also pointed out that methionine first influenced the bacteria with antioxidant functions.

Another group of researchers investigated the effects of a probiotic containing *Lactobacillus casei* LTL1879 as a single bacterial component on the organisms of participants. The results indicate a decrease in the MDA level and an increase in AOC, along with the absence of statistically insignificant changes in SOD activity. Along with these changes, there is a shift in intestinal flora. The expression levels of *Clostridium leptum*, *Bifidobacterium*, and especially *Lactobacillus* in the probiotic group were higher than in the control group after three weeks of administration. Moreover, modal testing demonstrated the positive correlations between *Lactobacillus* expression and SOD and AOC and the negative correlation between *Lactobacillus* expression and the MDA level [95]. Furthermore, *Lactobacillus* and *Lactobacillus plantarum* CCFM10 demonstrated similar effects. Aging modeling using D-galactose in mice caused the Firmicutes/Bacteroidetes ratio to increase, the relative abundance of *Lactobacillus* to decrease, and the growth of one genus of *Clostridiales*. Supplementation reversed these changes, as did the OS parameters. The authors assumed that the influence of *Lactobacillus* on the microbiome could be one of the mechanisms of OS regulation [19]. In addition to *Lactobacillus*, *Lonicera japonica* has also been investigated in an aging mouse model. *Lonicera japonica* polysaccharides influenced both systems by increasing the activity of antioxidant enzymes (SOD, CAT, and GSH-Px), reducing MDA concentration, and adjusting the Firmicutes/Bacteroidetes ratio along with increasing the relative abundances of Lactobacillaceae and Bifidobacteriaceae [1]. Supplementation of another lactic acid bacteria, *Pediococcus pentosaceus* ZJUAF-4, modulated the gut microbiome after toxic damage and improved the oxidative condition of the intestine [63].

Sesquiterpene glycosides from loquat leaf are potential probiotics that can cause OS to decrease and the ratio of Firmicutes/Bacteroidetes to decline, along with the relative abundances of Lactobacillaceae, Lachnospiraceae, and Muribaculaceae to increase [96]. A research team from China investigated the polysaccharides from *Chuanminshen violaceumin* in a naturally aging mouse model. The key points addressed in the article are the impact of the tested compounds on OS and inflammation in the liver and gut and its association with the gut microbiome. In addition to the evidence of alterations in bacterial community structure and improving the OS marker levels after supplementation, the authors presented data about correlations between the studied parameters. They stated that several genera (e.g., *Dubosiella*, *Lactobacillus*, *Parasutterella*, and *Limosilactobacillus*) were negatively correlated with the levels of MDA in serum, jejunum, and liver; by comparison, the other genera (e.g., *Staphylococcus*, *Acinetobacter*, and *Ligilactobacillus*) were positively correlated with the levels MDA in serum, jejunum, and liver. However, the correlation with the levels of antioxidant enzymes (CAT, SOD, and GSH-Px) was the opposite. The scientists suggested that intestinal flora mitigated OS and delayed aging through the gut–liver axis. They added that during aging, excess ROS led to OS, which caused intestine and liver damage, intestinal barrier damage, and microbiome disturbances [97]. The list of such compounds could be continued with taxifolin, which modulates intestinal flora and prevents OS and *Bacillus coagulans* XY2 [64]. In the Cu-intoxication model, taxifolin demonstrated abilities to mitigate oxidative damage by regulating intestinal flora, DAF-16/FoxO, and SKN-1/Nrf2 pathways [98].

Riboflavin (vitamin B2) is an important participant in many processes in organisms. The results showed that riboflavin supplementation modulates not only the OS parameters in serum but also in the colon. Vitamin B2 administration led to an abundance and diversity of gut microbiota, along with a relative upturn of *Prevotella* and *Absiella* and a decline in Proteobacteria, *Fusobacteria*, Synergistetes, and Cyanobacteria in strong conjunction with antioxidant properties. Moreover, the SCFA levels were also higher because of riboflavin supplementation [99].

Polyphenols are a group of phytochemicals with potential health-promoting effects. They are divided into two types: flavonoids (flavonols, flavanols, flavones, flavanones, isoflavones, and anthocyanins) and non-flavonoids (phenolic acids, hydroxycinnamic acids, lignans, stilbenes, and tannins) [100]. Flavonoids are compounds with diverse pharmacological effects. Many flavonoids influence the gut microbiome and OS. A natural flavonoid of the citrus species diosmetin in a colitis model demonstrated an antioxidant effect and changed the bacterial community structure in the gut. Diosmetin administration led to a decline in the abundances of *Eggerthella*, *Flavobacterium*, and *Clostridium* and the growth of Odoribacteraceae, *Prevotella*, Rikenellaceae, *Ruminococcus*, *Coprococcus*, *Roseburia*, *Oscillospira*, *Anaeroplasma*, and *Synergistales* compared with the colitis group. Moreover, the functional profile was also changed by the compound. However, the correlation between these two effects was not tested [36]. Furthermore, non-flavonoid molecules influenced these processes as well. OS was reduced, and the proportion of *Blautia* and *Dorea* (the butyrate producers) increased in the Lachaospiraceae family. In addition, *Bacteroides* and *Desulfovibrio* spp. associated with diseases and inflammation decreased. The authors mentioned that several OS parameters correlated with microbiome change [101]. Anthocyanins are water-soluble flavonoids ranging by nature, number, and location of carbohydrates attached to the molecule, the number of aliphatic or aromatic acids attached to them, the number of hydroxyls, and the degree of their methylation. Malvidin is one of the most well-known anthocyanidins, and this compound is commonly present in various fruits and vegetables [102]. The results of malvidin tests were similar to other flavonoids. Malvidin upregulated the antioxidant enzyme system and improved gut condition by affecting the relative abundances of Firmicutes, Bacteroidetes, and *Lactobacillus* [103].

A significant concentration of substances might improve people’s lives by mitigating the negative environmental influences and internal changes. The investigation of their methods of action could lead to identifying new possibilities to enhance quality of life (Table 3).

## 5. Discussion

OS is one of the key mechanisms in the development of many disorders [19,20,21,22,23,24], just as disturbances of the gut microbiome are associated with many and diverse human disease processes [13,28,29,30,31,32,33,34,35,36,48,49,50,51]. The OS–microbiome interaction might be relevant to understanding and correcting various conditions. One of the most popular directions of research in this field is the testing of substance impact on the oxidation system and human microbiome [64,93,94,95]. Food supplementation is a fast-growing area, and bioactive compounds are the core of this product line. Many different therapeutics prove their effectiveness every year, some of which have been used by humanity for centuries. Increasingly, the objective impact of these substances is being estimated. The accumulation of this information is important for determining the course of a more precise investigation. However, key concerns, such as the ways of interaction between the microbiome and OS, are not well addressed in many articles [36,61,96,97]. Although the aims of various types of research are finding correlations between the objects of study and changes in several systems in the organism, the authors quite rarely draw attention to the chemical characteristics or pharmacodynamics of the compound. Several hypotheses have been suggested, as shown in Table 4.

The first hypothesis is TMAO’s role in the microbiome–OS association [67,72,73,74]. Additionally, the interactions with mitochondria, including mitophagy regulation, are investigated [77,82]. The role of melatonin is discussed as well [34,35,91]. Another point that remains unclear is causation. Many compounds, including food supplements, could modulate oxidative balance and bacterial community [36,51,54,63,64,74,77,93,95,97]; however, these changes might be parallel processes or have a cause–effect relationship. The cause–effect relationships might be in two different directions and with different strengths. It should not go unnoticed that several researchers have analyzed the activation of the metabolic pathways, which could be involved in the studied processes [45,98]. These results are extremely important for theory development. The microbiome–OS association is a fast-growing research field; therefore, additional data on molecules and processes explaining the mechanisms of this link might be obtained in the near future.

## 6. Conclusions

There is much evidence for the existence of interactions between the gut microbiome and OS; however, the biochemical basis of such interaction is still unclear. Moreover, in certain pathological conditions, these processes might play an important role in the development of diseases. Although the features of the interaction between OS and the microbiome in the normal and abnormal changes and its contribution to disorders are analyzed in some studies, there are still many uninvestigated conditions and unexplored linker molecules. Since the main limitation of the studies in this area is finding correlations, instead of the mechanisms of the link, between OS and the microbiome, future research might aim to identify the molecules and processes of the interactions between these two systems. Furthermore, the subjects of research in this area are mainly animals. Future human studies might provide new information in this field.

## Figures and Tables

**Table 1:** SCFAs, OS, and the microbiome.

SCFAs	OS	Microbiome	Experiment	References
The levels of acetic and butyric acid were significantly lower, while propionic acid was undetected in the malathion group/control group. The levels of serum acetic and propionic acid significantly increased in the rifaximin group/malathion group. There were no significant differences in the butyric acid levels in the malathion group/malathion + rifaximin group.	The activities of SOD and CAT were lower, and MDA levels were higher in the malathion-treated group/control group. The activities of SOD and CAT significantly increased after rifaximin administration for two and four weeks in comparison with the malathion group, whereas the levels of MDA decreased after four weeks of rifaximin administration.	The main phyla in the gut microbiota were Bacteroidetes, Firmicutes, and Proteobacteria. The relative abundance of Firmicutes increased, and Bacteroidetes and Proteobacteria decreased in the malathion group/control group. Rifaximin treatment attenuated malathion-induced changes. The ratio of Firmicutes to Bacteroidetes increased in the malathion group/control group. It was mitigated by rifaximin treatment.	Disease: Testicular dysfunction Subjects: Rats Agent: Malathionan (an organophosphorus pesticide) Treatment: Rifaximin is a poorly absorbed antibiotic	[77]
The level was higher in the group without disease but not in the chitosan oligosaccharide group/control group. The level was higher in the group with disease and treated/group with disease and not treated.	Treatment decreased OS, showing a reduction in MDA levels and an increase in SOD levels.	The disease changes the Firmicutes/Bacteroidetes ratio. The treatment reversed it. The abundance of the probiotic family Muribaculaceae and genus *Akkermansia* rose after treatment. In the disease group, proinflammatory *Desulfovibrio* and *Dubosiella* increased. In the cohort with treated disease, it was lower. The same tendency was shown in *Escherichia–Shigella* and *Enterococcus*.	Disease: Acute pancreatitis Subjects: Mice Agent: Caerulein Treatment: Chitosan oligosaccharide administration	[32]
There was a significant decrease in butyric acid in the disease group/control group. The levels of acetic, propionic, butyric, isobutyric, valeric, and isovaleric acids were significantly improved in the treated group/disease group.	AOC and antioxidative enzyme activities, including GSH-Px, SOD, and CAT, significantly decreased, and the level of MDA and ROS were significantly higher in the disease group/control group. The SOD and CAT activities increased, and MDA and ROS levels decreased in the treated group/disease group.	The relative abundance of Bacteroidetes decreased in the treated group/disease group. The Firmicutes/Bacteroidetes ratio was significantly higher in the treated group/control and disease groups. *Bacteroides*, *Turicibacter*, Actinobacteria, Acholeplasmatales, *Eubacterium*, Staphylococcaceae, Enterobacterales, and Rhodespirillales predominated in the disease group. *Dubosiella*, Erysipelotrichales, *Desulfovibrionia*, Peptostreptococcaceae, and Patescibacteria predominated in the treated group.	Disease: IBD Subjects: Mice Agent: Dextran sulfate sodium Treatment: *Lonicera hypoglauca* and *Scutellaria baicalensis* as the beneficial bioactivities of extracts from *L. hypoglauca* and *S. baicalensis*	[33]

Abbreviations: Three blocks of investigated parameters are presented for each study, as well as the brief experimental conditions. SCFAs, short-chain fatty acids; SOD, superoxide dismutase; CAT, catalase; MDA, malondialdehyde; GSH-Px, glutathione peroxidase; AOC, total antioxidant capacity; ROS, reactive oxygen species; OS, oxidative stress; IBD, inflammatory bowel disease; and/, compared with.

**Table 2:** Melatonin, OS, and microbiome.

Melatonin	OS	Microbiome	Experiment	References
Per oral administration for three weeks.	The activity of SOD, CAT, and GSH-Px was higher in the control group/others and was lower in the condition group/treated group.	The ratio of Firmicutes to Bacteroidetes increased in the disease group/control group. Melatonin administration mitigated the changes.	Condition: Liver inflammation Subjects: Mice Agent: Ochratoxin A (the mycotoxin generated by *Aspergillus* spp.) Treatment: Melatonin	[34]
Melatonin supplementation.	There was a decrease in the CAT, GSH-Px, SOD, and AOC levels and an increase in the MDA level in the corticosterone group/control group. Sleep deprivation increased ROS production. ROS production in other groups showed no significant differences.	The ratio of Firmicutes/Bacteroidetes increased in the corticosterone group/control group. The relative abundances of Firmicutes and Proteobacteria increased, and the abundance of Bacteroidetes decreased in the corticosterone group/control group. After melatonin administration, it was mitigated. There was no statistically significant difference between the control group and the melatonin-supplemented group. The relative abundances of Firmicutes and Proteobacterium increased, and Bacteroidetes and Prevotellaceae decreased in the sleep deprivation group/control group. Melatonin pretreatment attenuated the effects of sleep deprivation on the intestinal microbiota disorder.	Condition: IBD Subjects: Mice Agent: Sleep deprivation or corticosterone supplementation Treatment: Melatonin	[35]
Supplementation with 10^−5^ mol/L of melatonin. The plasma melatonin level was decreased in the sleep restriction group/control group. No significant difference in melatonin was observed between the treated group and the control group mice.	The activities of antioxidant enzymes (GSH-Px, CAT, and SOD), AOC, and MDA content in the jejunum: The activity of enzymes and AOC were lower in the sleep restriction group/control group. The level of MDA was higher in the sleep restriction group/control group. No significant differences in AOC, MDA, GSH-Px, and CAT were observed between the treated group and the control group mice. SOD level was increased in the treated group/control group.	The relative abundances of Bacteroidetes and *Lactobacillus* were decreased, whereas that of Firmicutes, *Helicobacter*, and *Clostridium* and the difference in abundance between Firmicutes and Bacteroidetes were increased in the sleep restriction group/control group. Melatonin supplementation mitigated these patterns.	Condition: Sleep restriction Subjects: Mice Treatment: Melatonin	[91]

Abbreviations: Three blocks of investigated parameters are presented for each research, as well as the brief experimental conditions. Melatonin levels were not tested in several experiments; they were only administered. SOD, superoxide dismutase; CAT, catalase; MDA, malondialdehyde; GSH-Px, glutathione peroxidase; AOC, total antioxidant capacity; ROS, reactive oxygen species; IBD, inflammatory bowel disease; and/, compared with.

**Table 3:** Impact of food supplements on the gut microbiome and OS.

Compound	OS	Microbiome	Subjects	References
Proteins and amino acids	Proteins: No significant difference in MDA level between the two groups was detected. Methionine: Heavy metal exposure increased the intestinal MDA and decreased the antioxidant enzyme activity of GSH-Px, glutathione reductase, SOD, and CAT. MDA decreased in methionine-treated groups. Methionine significantly increased the content of antioxidative enzymes.	Proteins: The Bacteroidetes increased, and the Firmicutes decreased in the main group/control group. The presence of health-related taxa, including *Roseburia*, *Blautia*, and *Bifidobacterium longum*, was lower. Methionine: The abundances of Proteobacteria, Actinobacteria, Patescibacteria, and Cyanobacteria were higher in the methionine-treated group/control group.	Human Mice	[93] [94]
Probiotics (lactic acid bacteria)	*Pediococcus pentosaceus* increased the levels of SOD and GSH-Px. *Lactobacillus casei* LTL1879 increased the AOC level and decreased the MDA level.	The relative abundances of Firmicutes and Proteobacteria were increased in the disease group, which was reversed in the treated group. The decline in Bacteroidetes was restored by treatment. Supplement reversed the decrease in Muribaculaceae, Lachnospiraceae, and Defluviitaleaceae as well as the increase in Erysipelotrichaceae, Enterococcaceae, *Dubosiella*, and *Enterococcus* in the disease group. *Lactobacillus casei* LTL1879 reduced *Escherichia coli*, *Enterococcus*, and *Bacteroides* expression and increased *Clostridium leptum*, *Bifidobacterium*, and *Lactobacillus* expression.	Mice Human	[63] [95]
Other supplements	Sesquiterpene glycoside 3 was able to prevent oxidative stress. Taxifolin significantly mitigated oxidative stress injury by alleviating the levels of ROS and MDA as well as increasing antioxidant enzyme activity. *Bacillus coagulans* XY2 caused SOD, CAT, GSH-Px, and glutathione reductase to increase after Cu exposure.	Sesquiterpene glycoside 3 administration could decrease the ratio of Firmicutes/Bacteroidetes and increase the relative abundances of Lachnospiraceae, Muribaculaceae, and Lactobacillacea. Taxifolin treatment decelerated the D-galactose-induced aging process by regulating the composition of the intestinal flora and abundance of beneficial bacteria, including *Enterorhabdus*, *Clostridium*, *Bifidobacterium*, and *Parvibacter*. *Bacillus coagulans* XY2 reversed Cu and caused an increased level of *Enterorhabdus* abundance and decreased abundances of *Intestinimonas*, *Faecalibaculu*, Ruminococcaceae, and Coriobacteriaceae*_UCG-002*.	Mice	[64,96,97,98]
Riboflavin	Supplement elevated CAT, SOD, AOC, and decreased MDA level in the serum.	The relative abundances of *Prevotella* and *Absiella* were higher, and the relative abundances of *Proteobacteria*, *Fusobacteria*, *Synergistetes*, and *Cyanobacteria* were lower in the supplemented group.	Mice	[99]
Polyphenols	MDA level in colon tissues was decreased, and the levels of SOD, GSH, and GSH-Px were increased by diosmetin. Sinapic acid consumption decreased ROS and MDA levels in the colon and increased AOC in the liver. Malvidin anthocyanins increased the activities of SOD, GSH-Px, CAT, and AOC but decreased the levels of MDA in the serum and liver.	The diosmetin-treated group had increased relative abundances of Bacteroidetes and Cyanobacteria and a decreased relative abundance of Firmicutes compared with the disease group. The abundances of *Eggerthella*, *Flavobacterium*, and *Clostridium* were lower, and the abundances of Odoribacteraceae, *Prevotella*, Rikenellaceae, *Ruminococcus*, *Coprococcus*, *Roseburia*, *Oscillospira*, *Anaeroplasma*, and *Synergistales* were higher in the diosmetin-treated group/disease group. Polyphenol supplementation (resveratrol and sinapic acid) increased the proportion of butyrate produced in the Lachaospiraceae family and inhibited the growth of bacterial species associated with diseases and inflammation such as *Bacteroides* and *Desulfovibrio* spp. Malvidin anthocyanins increased the relative abundance of Firmicutes and decreased the relative abundance of Bacteroidetes.	Mice Rats	[36,103] [101]

Abbreviations: Two blocks of investigated parameters are presented for each research, as well as the supplement type. SOD, superoxide dismutase; CAT, catalase; MDA, malondialdehyde; GSH-Px, glutathione peroxidase; AOC, total antioxidant capacity; ROS, reactive oxygen species; GSH, glutathione; and/, compared with.

**Table 4:** Possible interconnections between OS and the microbiome.

Agent	Possible Mechanism	References
TMAO	An imbalance in the microbiome composition; precisely, *Bifidobacterium* abundance declines and Firmicutes/Bacteroidetes ratio changes, which causes a rise in TMAO level, which, in turn, leads to OS.	[74]
SCFAs	Several SCFAs might modulate mitochondrial function, including oxidative processes. The gut microbiome may influence autophagy processes by producing SCFAs. The damaged mitochondria generate excess ROS, and their autophagy leads to decreased OS in cells.	[1,77]
Melatonin	Melatonin alters colonic microbial composition and diversity, resulting in decreased Gram-negative bacterial-derived lipopolysaccharides that could enter into the blood and liver via defects in the intestinal barrier. These effects lead to the alleviation of ochratoxin A-induced liver inflammation, oxidative stress, and mitophagy.	[34]
Genes	It was suggested that high *MUC1* (OS causal gene) expression accompanied by decreased production of microbiota-derived metabolites could confer an increased risk of CD. CD40 and its ligand (CD40L) are associated with ROS production. It was suggested that genetic variants regulate CD40 expression and interact with inflammation-related microbial activities and, therefore, contribute to CD pathogenesis through three microbial pathways associated with *CD40* gene expression: nicotinamide adenine dinucleotide biosynthesis from aspartate, L-isoleucine biosynthesis, and the superpathway of pyridoxal 5-phosphate biosynthesis and salvage. PRKAB1 expression may be negatively associated with the risk of developing CD because of the interaction between gene expression and intestinal microbial nucleotide metabolism.	[45]
Supplements	No mechanisms were presented in this study. The influence of supplements could be parallel or co-dependent.	[19,36,63,64,74,97,98,101,103]

Abbreviations: Suggested mechanisms for each discussed interconnection are presented. TMAO, trimethylamine-N-oxide; OS, oxidative stress; SCFAs, short chain fatty acids; ROS, reactive oxygen species; *MUC1*, hypoglycosylated abnormal mucin 1; CD, Crohn’s disease; and PRKAB1, protein kinase amp-activated beta 1.
